# Feasibility, acceptability, and fidelity evaluation of a contextualised physical activity and diet intervention for hypertension control in rural South African adults

**DOI:** 10.1371/journal.pone.0348696

**Published:** 2026-05-08

**Authors:** Kganetso Sekome, Francesc Xavier Gómez-Olivé, Hellen Myezwa, Dale W. Esliger, Lauren B. Sherar

**Affiliations:** 1 Department of physiotherapy, School of Therapeutic Sciences. Faculty of Health Sciences, University of the Witwatersrand, Johannesburg, South Africa; 2 School of Sport, Exercise and Health Sciences, Loughborough University, United Kingdom; 3 MRC/Wits Rural Public Health and Health Transitions Research Unit (Agincourt), School of Public Health, Faculty of Health Sciences, University of the Witwatersrand, Johannesburg, South Africa; 4 School of Therapeutic Science, Faculty of Health Sciences. University of the Witwatersrand, Johannesburg, South Africa; Tribhuvan University Institute of Medicine, NEPAL

## Abstract

Despite the known benefits of physical activity and diet modifications for hypertension control, adults in rural South African settings still have high levels of uncontrolled hypertension. This paper outlines an intervention that targets adjusting routine physical activity and diet practices for hypertension control in adults from rural South Africa (HYPHEN). The intervention involved a structured group education, individualised physical activity education, and individualised dietary education. We aimed to evaluate the feasibility, acceptability, and fidelity of HYPHEN for adults aged 40 years and older living with hypertension in rural South Africa. Feasibility was measured by assessing recruitment and retention rates. Acceptability was assessed through interviews after the intervention using pre-determined themes of perceived expectations, benefits, motivation, and barriers concerning the intervention. Fidelity was evaluated by intervention adherence, dosage, quality, and participant responsiveness. Participants were also asked to rate their overall satisfaction on a Likert scale out of 10. Our study demonstrated high level of feasibility, acceptability, and fidelity. Thirty participants were successfully recruited (100% of target), 28 (93%) participants were retained, and 28 (93%) provided complete data. Qualitative data demonstrated high acceptability, with participants reporting that the intervention met expectations, provided benefits, motivated continued engagement, and involved few barriers. Intervention fidelity was high with all three components carried out as planned, minor dosage adjustment, high delivery quality, and 100% participant engagement. The average rating on the Likert scale was 8.6/10 (SD = 2.55). HYPHEN is a feasible and acceptable intervention for hypertension control. Trial registration: Pan African Clinical Trial Registry (pactr.samrc.ac.za) PACTR202306662753321. Retrospectively registered on 29 June 2023.

## Introduction

About one in three Africans has hypertension, with its prevalence varying across various regions of the continent and among different age groups [1]. The highest reported prevalence of hypertension was in South Africa (41.6% − 54.1%), while the lowest was in Burkina Faso (15%) [2]. The highest prevalence was reported for individuals aged >75 years [1]. Hypertension prevention and control efforts vary globally; however, a consistent pattern of higher hypertension prevalence is observed in low- and middle-income countries (LMICs) compared to high-income nations [3]. This disparity is largely driven by persistently low rates of hypertension detection and treatment in Africa [4]. The World Health Organization (WHO) African Region is reported to have the highest prevalence of hypertension, whereas the WHO Region of the Americas reports the lowest [5]. Hypertension is a modifiable risk factor that can be controlled through lifestyle changes.

The benefits of physical activity and dietary modifications for hypertension control have been well documented [6–8]. Evidence suggests that adults with chronic diseases can benefit from increasing their physical activity, including increasing the number of steps taken per day [9], increasing the intensity of daily physical activity [10], or simply participating in regular physical activity according to their level of ability [11]. The widely recommended dietary approaches to control hypertension are through the reduction of dietary sodium and adoption of the Dietary Approaches to Stop Hypertension (DASH) diet [12]. The DASH diet recommends that people with hypertension consume vegetables, fruits, and whole grains while limiting sodium, saturated fat, and added sugars.

Although physical activity and dietary changes are globally recognised as effective for controlling hypertension, adults in rural South Africa continue to experience high rates of uncontrolled hypertension due to limited local adoption and contextualisation [13]. Recent data from rural north-east South Africa shows that 58.7% of adults aged 40 years and above have uncontrolled hypertension [13]. Some of the identified challenges for the low control rates in rural South Africa include inadequacies of the healthcare system, affordability issues with the recommended diet, and a poor understanding of the daily physical activity routine of the population [14,15]. Other challenges which have been reported globally include low frequency of interaction with health staff, (un)availability of clear clinical guidelines, and the unavailability of medicine [16]. The low rate of hypertension control for adults in a rural population contributes to economic hardships due to the associated costs of accessing the healthcare system, low doctor-to-patient ratios, and lack of access to health information [17,18].

A low doctor-to-patient ratio means there are fewer opportunities for early diagnosis, regular checkups and access to treatment for hypertension. People with hypertension often go unmonitored for long periods, which increases the risk of complications like stroke, heart attack, or kidney failure [19]. The impact on the health system and the individual includes higher medical costs for emergency care, hospitalisation, medications, and potential long-term disability. Lack of access to health information often results in the use of ineffective or harmful traditional remedies, poor medication adherence, resulting in disease progression and more costly interventions later [20].

Interventions at the individual level, which do not require financial commitment from the hypertensive individual, can substantially reduce the burden of hypertension on the individual [21]. A review by Turkson-Ocran RAN and colleagues [22] highlights the importance of addressing social determinants of health in hypertension management. The review encourages community interventions for lifestyle modification, such as culturally-tailored health education, engaging community health workers, and collaborative care models linking clinics and communities. This type of approach has not been fully explored in the South African rural population [23].

In line with the WHO recommendations for lifestyle approaches to control hypertension through physical activity and diet [6], rural populations require affordable and/or free tailored interventions that suit their contextual needs. Factors to consider when contextualising interventions for rural populations include the social, cultural, geographic, and economic needs of that population, as was considered in our proposed intervention. Existing interventions do not consider the cultural and social beliefs and practices that influence behaviour change for diet and physical activity in a rural population, hence poor hypertension control.

Adults in rural South Africa have been reported to engage in various daily physical activity routines such as walking as a means of transportation, walking for wood and water collection, yard work, various aspects of farming, and housework [24,25]. These daily routines are, however, performed at intensities that may not be sufficient to achieve health benefits [6,26]. To gain health benefits from physical activity, the intensities performed should aim to increase the individual’s heart rate, causing vasodilation, and resulting in better vascular function [27]. For adults in a rural population, the intervention should focus on increasing the intensity of already existing daily physical activity routine [26]. The WHO recommends physical activity of moderate to vigorous intensities for health benefits from chronic conditions such as hypertension [28].

The majority of published dietary interventions involve food recommendations which are unaffordable in many rural communities [29]. Considering these exisiting knowledge, the contextualised HYPHEN (**HY**pertension control using **PH**ysical activity and di**E**t in a rural co**N**text) intervention was developed based on the social and cultural beliefs, perceptions and practices regarding physical activity and diet from hypertensive adults in rural South Africa [30]. The aim of the present study was to evaluate the feasibility, acceptability, and fidelity of HYPHEN for adults in rural South Africa. We sought to assess feasibility by evaluating participant recruitment, retention, completeness of data. Acceptability and satisfaction were evaluated using in-depth interviews post intervention and Likert Scale rating. We assessed fidelity of intervention delivery by evaluating intervention adherence, dosage, quality of intervention delivery, and participant responsiveness.

## Methods

The full protocol for the HYPHEN study has been published elsewhere [30]. We recruited 30 adult participants with a self-report hypertension diagnosis between 10/04/2023 to 11/04/2023. Feasibility studies do not require a formal power calculation or hypothesis testing as the aim is not to assess intervention effect [31]. Primary outcomes were feasibility, acceptability, and fidelity of delivery.

### Study design

The study employed both quantitative and qualitative methods. The quantitative part consisted of a single-group, community-based evaluation examining the feasibility of a 10-week, contextually tailored physical activity and diet intervention for adults with hypertension. The qualitative part involved conducting in-depth interviews with participants enrolled in the intervention. Inclusion criteria included adults aged 40 years and older; a self-reported diagnosis of hypertension and/or current use of medication for hypertension; and residency in the study area for at least six months prior to the intervention start. We excluded participants who are using mobility aids as this would affect accurate physical activity measurements using accelerometers.

### Setting and population

The study was conducted at Bushbuckridge sub-district in Mpumalanga Province of South Africa where the Agincourt Health and Demographic Surveillance System (HDSS) has been running since 1992. Participants were sampled from the Health and Aging in Africa: A Longitudinal Study of an INDEPTH (International Network for the Demographic Evaluation of Populations and Their Health) community in South Africa (HAALSI) [32]. Participants were contacted via telephone and given a description of the study. Those who expressed interest to participate were then visited at their households for further explanation and to obtain written informed consent to participate.

### Ethics approval and consent to participate

This study required clearance from two ethical bodies. The first clearance was obtained from University of the Witwatersrand Human Research Ethics Committee (HREC – Medical) (clearance number: M 210282). The second clearance was obtained from the local Provincial Department of Health Research and Ethics Committee (clearance number: MP_202106_001). Consent considerations and confidentiality of all research data was adhered to. This study is based on the usual ethical principles, such as every person’s right to refuse to participate in the study and to withdraw at any time, as well as respect for all participants and protection of their privacy.

### Intervention procedure

Three locally trained fieldworkers, who are residents of the study area and native speakers of Xitsonga, delivered and assessed the intervention. The fieldworkers were trained by the first author, including questionnaire administration, anthropometric measurements, and delivering education to the participants.

### Intervention

The intervention comprised three components: structured group education, individualised physical activity education, and individualised dietary education and is described in full elsewhere [30]. The intervention was designed following the Intervention Mapping protocol, a planning approach to develop theory- and evidence-based health-promoting interventions [33]. We also used the COM-B model of behaviour change as well as taxonomies of behaviour change developed by Michie and colleagues [34,35] to design the intervention. Continuous consultations with study participants and topic experts were used to co-design the intervention. The intervention was delivered in Xitsonga, which is the participants’ home language. The structured group education focused on enhancing participants’ knowledge and skills by providing explanations about hypertension, its related risk factors and its management. Information pamphlets were given to each participant. Individual household visits were conducted to impart behaviour change techniques that target participants’ individual physical activity and diet. As part of the behaviour change strategy, participants were asked to set individual goals that they wanted to achieve over ten weeks. Participants were provided with individualised education on how to make modifications to their daily physical activity and dietary routine. Monitoring of behaviour change and feedback was conducted by the field-workers via weekly telephone calls to monitor participation and engagement throughout the ten-week intervention. The first author was in contact with field-workers telephonically every week for the ten weeks to discuss outcomes of participation. There was no compensation or incentive provided for being part of the intervention, because there was no financial or additional time commitment required from participants for being part of the intervention. Study participants were also informed that they would not be provided with any resources to supplement their physical activity or diet.

### Data collection method and outcomes

The outcomes of interest were the intervention’s feasibility, acceptability, and fidelity. We used the tips recommended by Avery and colleagues [36] to judge the feasibility of progressing to a larger scale evaluation. This comprised of successful recruitment of 30 participants in one week, ≥ 80% of recruited participants retained, ≥ 80% of recruited participants provide completed weekly data. Acceptability was assessed through interviews at the end of the 10 weeks intervention. The interviews explored participants’ perceived expectations, benefits, motivation, and barriers concerning the intervention. Overall satisfaction was rated using a Likert Scale with a rating of 7/10 being considered acceptable satisfaction. Fidelity of the delivery of each component of the intervention was assessed by the primary investigator using the five dimensions of fidelity, namely: adherence, dosage, quality of intervention delivery, participant responsiveness, and program differentiation [37]. We assessed all but program differentiation. The reason was because our intervention consisted of a one arm group with no control group to compare differences.

### Quantitative analyses

**Fig 1:**
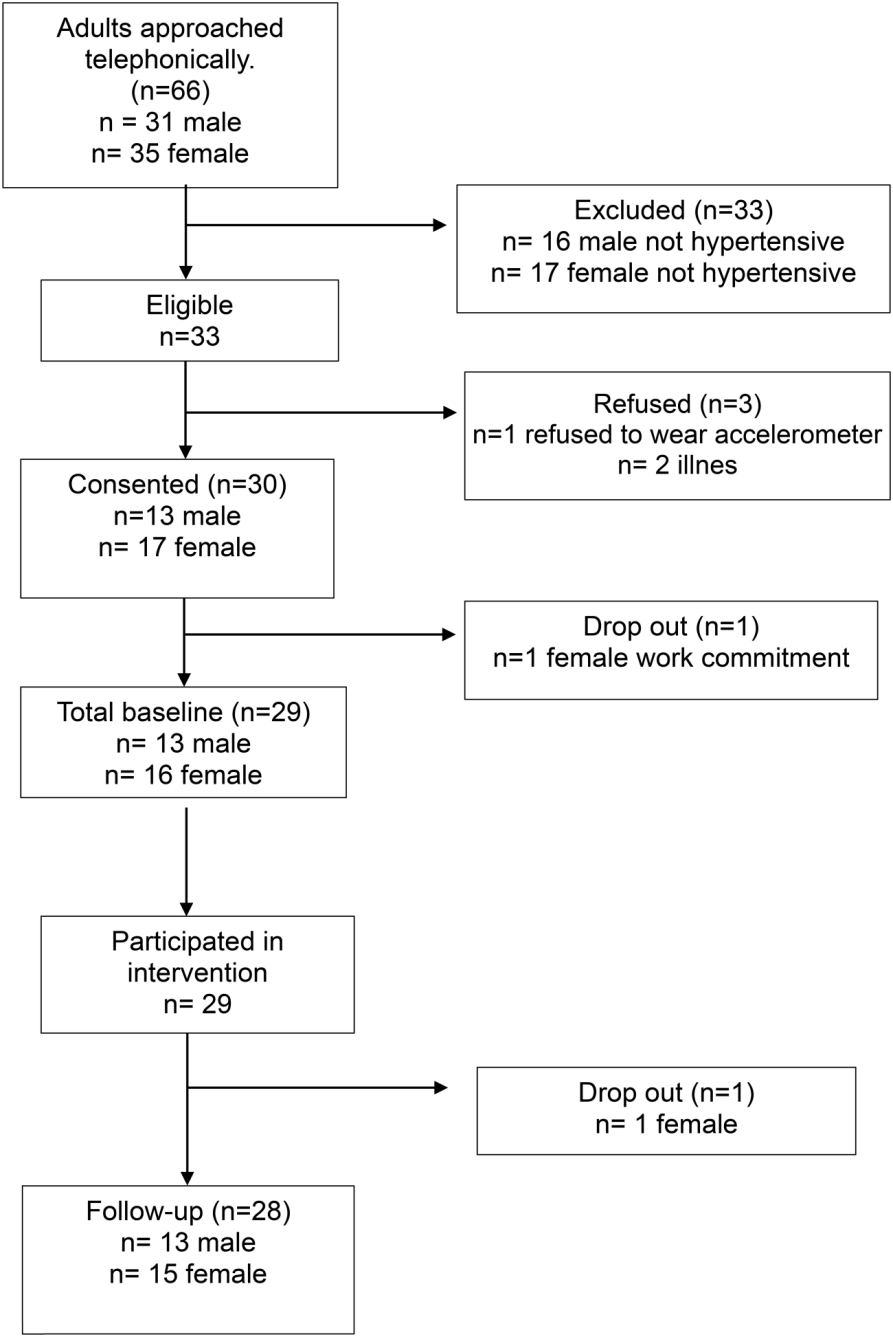
CONSORT diagram.

A Consolidated Standards of Reporting Trials (CONSORT) flowchart was used to present the flow of participants (Fig 1). Descriptive statistics of means, standard deviations, percentage, and proportions were used to measure outcomes of feasibility. All data were aggregated into REDCap. No further statistical testing was measured as the primary outcomes were feasibility and acceptability without any assessment of effect.

### Qualitative analysis

Audio-recorded interviews were transcribed and translated from Xitsonga to English by two locally trained, qualitative research assistants. Transcripts were analysed manually by the principal investigator for themes relating to the acceptability of the intervention guided by Braun and Clarke’s approach to thematic analysis [38]. All co-authors confirmed the codes and themes. Coding was performed deductively according to already pre-determined themes of: perceived expectations, benefits, motivation, and barriers of the intervention [39]. “Open coding” was performed which is a process of labelling text to identify concepts related to acceptability. The process enhances rigor by allowing for different views. All co-authors confirmed the final codes.

## Results

### Participant recruitment

The CONSORT diagram, which presents the flow of participants through the intervention, is presented in Fig 1. We generated a random sample of previously confirmed hypertensive participants from the HAALSI study to obtain the required sample. We phoned 66 participants, and 33 (50%) were eligible to participate in the study—all adults who were ineligible reported not having hypertension. Although not explicitly assessed, the refusal of a hypertension diagnosis may be attributable to factors such as perceived stigma or a lack of interest in participating in the study. Of the eligible participants, three declined to participate due to illness (n = 2) and unwillingness to wear an accelerometer (n = 1). A total of 30 participants were recruited via telephone over two days. One participant contacted the research team three days after recruitment and requested to withdraw due to moving out of the study site for work. A total of 29 (97% of those recruited) participated in the intervention and had complete baseline data. One participant dropped out during week 10 due to spousal death and could no longer engage with the research team for follow-up assessments due to cultural reasons. In total, 28 (93%) of the 30 recruited participants completed the intervention and had follow-up assessments for ten weeks. Their characteristics are presented in Table 1.

**Table 1:** participants’ characteristics.

Variable	Baseline (N = 29)	Follow-up (N = 28)
Sex		
Male	13 (45%)	13 (46%)
Female	16 (55%)	15 (54%)
Age		
50-59	10 (35%)	10 (36%)
60-69	12 (41%)	11 (39%)
70+	7 (24%)	7 (25%)
Employment status		
Employed	1 (3%)	1 (4%)
Unemployed	28 (97%)	27 (96%)
Educational level		
Primary education	19 (66%)	18 (64%)
Secondary education	10 (34%)	10 (36%)
Alcohol drinkers	3 (10%)	3 (11%)
Tobacco smokers	2 (7%)	2 (7%)

### Participants’ characteristics

The baseline sample (n = 29) was female (16, 55%), unemployed (28, 97%), lower educated [attended only primary school (19, 66%)], non-smokers (27, 93%), and non-alcohol drinkers (26, 90%). The mean age was 64.9 years. One participant did not complete the follow-up assessment.

### Outcomes

#### 1. Feasibility.

A pre-defined progression criteria [36] outlined in the study protocol [30] was used to judge feasibility of progressing to a larger intervention (Table 2). The criteria are also outlined using the traffic light system, where Green represents the intervention can proceed to a larger trial with possible minor amendments, Yellow represents the intervention should undergo moderate amendments before a larger trial, and Red means the intervention should not proceed until major changes occur. We recruited 100% participants in two days, 93% of participants were retained to completion, 93% of participants engaged with weekly telephonic calls, and measurements of all outcome data of feasibility were complete.

**Table 2:** HYPHEN progression criteria.

HYPHEN progression criteria	Go – proceed to larger scale (RCT)	Amend – Proceed with changes	Stop – Do not proceed unless changes possible	HYPHEN results
Feasibility of participant recruitment – can 30 participants be recruited in one week?	If 30 participants are recruited in one week (100%)	If 15–29 participants are recruited in one week (50 to <100%)	If <15 participants are recruited in one week (<50%)	30 (100%) participants in 2 days
Feasibility of patient retention – can at least 80% of recruited participants be retained?	≥24 (80%) retained	21-24 (70–80%) retained	<21 (70%) retained	28 (93%) participants retained
Feasibility of engagement – can at least 80% of recruited participants provide complete weekly data	≥24 (80%) engaged	21-24 (70–80%) engaged	<21 (70%) engaged	28 (93%) participants engaged.
Feasibility of intervention implementation	Delivery of intervention judged strongly feasible by qualitative data	Delivery of intervention judged feasible by qualitative data	Delivery of intervention judged possibly feasible by qualitative data	Participants quotations very positive.

#### 2. Acceptability.

Five pre-determined themes were used to judge the acceptability of the intervention: [1] meeting intervention expectations, [2] benefits of the intervention, [3] motivators to continue with intervention, [4] barriers associated with the intervention, and [5] suggestions for improvement.

#### 2.1 Meeting intervention expectations.

Most participants expected a reduction in blood pressure from the intervention and they believed that this was achieved but only modestly. However, some participants mis-understood the intervention and expected that the intervention would also provide them with anti-hypertensive pills, food parcels and money to buy healthier food.

- *“Yes it did (meet my expectation) because the level my high blood pressure had decreased a little. I want you to keep on doing what you are doing to us because we are now living good without any complications”.* Female, aged 57.
- *“No, they did not reach my expectation. I was expecting [removed] to give me pills after screening me and find that I have high blood pressure and I was also expecting that they will give us some food parcels”.* Female, aged 71.
- *“If you were offering us the food that is in the diet maybe things would be easy for us to stick to the diet. For now, the best thing is not to eat salted food and to eat brown bread because it is something that we can do simple”.* Male, aged 70.

#### 2.2 Benefits of the intervention.

Some of the intervention benefits expressed by participants included: reduced night sweats, awareness of blood pressure reading, reduced heartbeat, improved daily activity performance, greater family involvement in health management, overall health improvement, no body pains, and reduced shortness of breath.

- ***“****my high blood pressure is no longer troubling me like it used to, I would sweat a lot at night when I am sleeping but now I don’t*”. Male, aged 68.
- *“I did not even know my high blood reading but they (field workers) were able to alert me about it.” Female, aged 68.*
- *“Even my heart beating is no longer that fast as it used to be”.* Female, aged 68.
- *“Taking a long walk was difficult for me but now I am able to take a walk from here in my household to Newington and I can also play with children as well”.* Female, aged 68.
- *“Those that are cooking for us now know that they don’t have to add more salt on our food but a small amount of salt because they know that you told us to follow the diet”.* Female, aged 57.
- *“It took me days to get used to the food but now I am used to it and I am even complaining if they had added much salt on the food”.* Male, aged 70.
- *“I used to sweat at night when I am asleep, felling pains all over my body and struggling to walk around but ever since with the intervention of diet and physical activity I am able to do a lot of things and I can feel it in my body that I am fine now”.* Female, aged 52.

#### 2.3 Motivators to continue with the intervention.

The participants were motivated to continue with the intervention because they saw a positive health change, were encouraged by receiving weekly calls from field workers, and they had hopes of feeling better as a result of the intervention.

- *“What gave me energy is the education that I got from you, and I was able to reduce salt and also eat fruits and vegetables that I was told to eat. That is what motivated me because I could see a change about my health”.* Female, aged 68.
- *“The phone calls motivated me, because you always call me to check what have I eaten and you also ask that did I do any activities? So that is encouraging us to do it every day”.* Female, aged 68.
- *“What motivated me was the advices, the education you gave us, it gave me hope that if I follow your instructions I will be fine”.* Female, aged 52.

#### 2.4 Barriers associated with the intervention.

Some of the challenges expressed by participants during the intervention included difficulty adapting to the recommended diet due to learnt habits (e.g., high salt food intake) and difficulty measuring how much salt to add when cooking for household. The participants alluded to the fact that the diet recommendations were clear but they required a longer duration for them to adapt to making changes in their diet. For physical activity most participants reported no barriers as they said the intervention was embedded into their daily routine activities they were already performing.

- *“When it comes to diet it was very difficult because I was used to eating whatever I want but when I was taught about good diet I had to change and it was difficult, I am doing it because I want my health to be good and my blood pressure to reduce”.* Male, aged 67.
- *“I only had a challenge with salt measurements in food but the rest I did not have any challenges”* Female, aged 68.
- *“There were no challenges because something like going to the field and cleaning my yard is daily routine so I did not have any problem by doing it”.* Female, aged 57.

#### 2.5 Suggestions for improvement.

Suggestions given to improve the intervention included the provision of healthy food, starting a community farming project, the provision of blood pressure machines, and expanding the intervention to more people in future. Participants were also asked to rate their overall satisfaction on a Likert scale out of 10. The average rating on the Likert scale was 8.6/10 (SD = 2.55). The lowest participant rating was 6 (n = 4) while the highest rating was 10 (n = 12). This demonstrated that the intervention was acceptable.

- *“I rate it 8 to 10 because when there are people who are always reminding you about your health it is good. I used to chase [removed] people out of my home but now I welcome them with open arms”.* Female, aged 73.
- *“I would rate it 10, the intervention was great to me. I want the intervention to continue helping our society and I am very much happy with it”.* Female, aged 52

#### 3. Intervention fidelity.

- *“If it was possible, you must open a project where we can go and do our farming where we will plough vegetables like spinach and pumpkins that will always keep us healthy”.* Female, aged 68.
- *“You should give us these (blood pressure) machines because if you take them with you we are not able to know if the level of high blood pressure is stable or I am in danger”.* Female, aged 57.
- *“You must continue to remind us about doing exercises and also motivate us because if you don’t do that we will stop as time goes on”.* Male, aged 67.
- *“You had given us the perfect diet so I don’t think you should add on it or remove something, just keep on implementing it on other participants.* Male, aged 64.

All four assessed dimensions of fidelity demonstrated that the intervention was delivered as intended. All three intervention components were delivered as planned with full participant adherence. The group educational sessions were conducted within the expected timeframe, with field workers adhering strictly to the printed educational materials, and all participants demonstrating consistent responsiveness to the weekly telephonic follow-ups. Table 3 outlines the outcomes of the intervention fidelity.

**Table 3:** Fidelity of delivery of intervention.

Measure of fidelity [37]	HYPHEN study fidelity outcome
**Adherence**	The three intervention components—group-based education, individualized physical activity, and individualized dietary education—were implemented as planned. All participants attended the sessions, adhered to weekly monitoring telephone calls, and received household visits at the conclusion of the intervention. During each weekly call, the research assistants asked participants which components of the intervention were achieved for that week and ticked this on the data collection sheet.
**Dosage**	The group educational interventions were planned for 90 minutes, but could be slightly shorter or longer by 15 minutes, depending on the level of engagement and participation. They lasted between 75–100 minutes. This showed good fidelity.
	Individual educational interventions were planned for 60 minutes but could be slightly shorter or longer by 10 minutes depending on the participant’s set goals, they lasted between 60–70 minutes which showed good fidelity.
**Quality of intervention delivery**	Field workers had copies of educational material that they followed for each of the three interventions. There was no deviation from the planned educational material content. The primary researcher who designed the intervention materials was present throughout all interventions to assess the extent to which the materials were delivered as planned and note any deviations.
**Participant responsiveness**	Participants were telephoned weekly to assess engagement with the intervention. Field workers kept record of participants who refused to engage. 100% participants were called weekly and 100% engaged with the calls, which lasted between 10–20 minutes.

## Discussion

Our study aimed to evaluate the feasibility, acceptability, and fidelity of HYPHEN, a contextually relevant physical activity and dietary intervention for controlling hypertension in adults aged 40 years and older living in rural north-eastern South Africa. Our study demonstrated that HYPHEN was feasible and accepted. The design and methods were feasible, as evidenced by recruitment within the set time frame, high intervention adherence, engagement, retention, and positive feedback during qualitative interviews. The mean score for intervention satisfaction was a Likert scale of 8.6/10. The intervention also demonstrates a promise of success if rolled out on a larger-scale trial. Almost all our study participants reported being unemployed. Trends of rural employment in African countries indicate high unemployment in rural communities, with the majority of adults depending on agricultural and environmental landscapes for self-employment [40]. It is possible that some of these participants had self-employment, an aspect we did not assess. The gender distribution was almost equal; however, many of the participants only had primary education as their highest level of education. The low education level can be linked to the mean age of participants in our study which was 64.9 years. Rural dwellers are more likely to be older with a lower level of education than urban dwellers [41].

### Feasibility

During participant recruitment, half of the adults we approached telephonically did not disclose to our fieldworkers their hypertension status, although we recruited a sub-sample of participants in the HAALSI cohort (adults 40+) who had been found to be hypertensive in the last cohort visit in 2022 [13]. It is possible that the HAALSI study might have wrongly identified some participants as hypertensive, but they were not confirmed as hypertensive when they attended the local health facilities. This points to the limitations in self-reported hypertension diagnosis without a confirmed measurement as well as the use of field workers who may not be adequately trained for health measurements. Also, due to its asymptomatic nature, most adults who have been newly diagnosed with hypertension may not be aware of being hypertensive. A study in Nepal reported that hypertensive patients who do not experience dizziness and headaches perceive their blood pressure to be normal [42]. Findings from our study highlights the need to improve objective screening of hypertension at the community level and to increase education around hypertension. Efforts to achieve these are ongoing [13,43–45]. It is important to consider that some participants may have denied having hypertension due to perceived stigma or may have declined participation because of concurrent involvement in multiple other studies. Besides the denial of diagnosis, only one female participant refused to take part in our study because she did not feel comfortable wearing an accelerometer device on her wrist due to the potential for skin irritation. However, it has been previously reported that compliance with wrist-worn accelerometers is high due to lower participant burden and reduced potential for skin irritation [46].

Our study demonstrated strong indicators of feasibility as it met the criteria for “Go – proceed to larger scale” in all four areas. Our recruitment reached 100% of the target sample within two days, which substantially exceeds typical benchmarks reported in similar feasibility trials [39,47]. Particiants’ retention also surpasses the commonly cited 70–80% threshold which is considered acceptable in feasibility research. Participants’ engagement during telephonic calls indicated strong adherence to the delivery model. Moreover, the completeness of outcome data across all feasibility measures minimises uncertainty regarding trial procedures and strongly supports confidence in data collection method practicality for a larger trial. Our study lost one participant due to migration for employment at a different community outside our study site. This is not a unique phenomenon because migration out of rural communities in South Africa is very common due to people always seeking improved livelihoods [48,49]. We lost another participant during week nine of the intervention due to cultural reasons, as her husband had passed away. It is expected for women in some rural South African traditions to socially exclude themselves from society for several weeks to months as part of the mourning period [50], therefore, we could not engage with the participant for final follow-up assessments. Beyond procedural success, our study’s rapid recruitment and sustained engagement reflects a strong contextual relevance and low participant burden; factors which have been linked to successful progression from feasibility to definite trials [47,51].

### Acceptability

Participants in our study expressed multiple expectations from the intervention during follow-up interviews. Their main expectation was a reduction in elevated blood pressure, which was modestly achieved. Other expectations were a supply of antihypertensive medication, healthier food parcels, and money to purchase food options that are low in sodium salt. It was not the intention of our intervention to provide participants with any supplies of medication, money, or food parcels. Due to multiple previous and ongoing studies at the study site, participants have been exposed to other studies that have provided incentives; this could have created the expectation that we would also provide incentives. A future, larger-scale intervention should make it clear to participants what the intervention will not provide to avoid wrong expectations that could affect participation in the study. An intervention conducted in the same study site for adults aged 60 years and older showed that a monthly provision of low-sodium salt contributed to the reduction of hypertension, especially for participants who struggled to access fruits and vegetables [44]. It was not the primary aim of our study to measure changes in blood pressure, but it is noted that hypertensive individuals can benefit from the provision of food and equipment supplies that can assist in blood pressure monitoring and control, a consideration for a future clinical trial. Salt reduction is essential for controlling blood pressure [12]. Future interventions should promote long-term use of affordable low-sodium salt, particularly in rural South Africa, where regulation of Sodium in processed food is poorly monitored [52] and much of the salt intake comes from salt added during cooking or at the table. [53].

Our study participants found the intervention acceptable because they perceived a reduction in high blood pressure-related symptoms. Participants reported reductions in night sweats, palpitations, generalised body pain, and dyspnoea. Contemporary evidence indicates that when patiens perceive symptomatic improvement or measurable health gains, they are more likely to adhere to and complete behavioural interventions. For example, improved symptom appraisal has been linked to better adherence and sustained engagement in chronic disease programmes in studies by Burnier & Egam [54]. These findings underscore the importance of incorporating patient-reported outcome measures in hypertension intervention, as perceived benefit may be a critical mediator of acceptability and longer-term adherence. A scaled-up HYPHEN intervention must systematically document participants’ clinical histories [55]. Beyond the clinical symptom benefits, participants in our study also described increased awareness of their blood pressure readings and greater family involvement in health management. Recent literature highlights that self-monitoring, family, and social support are associated with improved blood pressure control and sustained self-management [56,57]. Collectively, our findings suggests that the intervention’s acceptability was not only driven by symptomatic relief but also by enhanced hypertension literacy, self-efficacy, and social support. These factors are central to effective long-term hypertension control.

Our findings that study participants faced challenges in adapting to recommended dietary changes—particularly due to long-standing habits of consuming high-salt foods and difficulty in knowing and measuring appropriate salt quantities when cooking for their households—corroborate previous findings reported in other settings [58]. There is limited research that shows the duration of habit formation related to dietary changes and physical activity, however, studies suggest that eating habit formation is estimated to take between two to eight months, with 66 days being an average [59]. We suggest that a future larger-scale intervention considers prolonging the intervention to allow for participants to adapt and sustain the recommended changes in diet. Regarding changes in physical activity performance, most participants reported no barriers because the intervention was embedded into their daily routine activities. Our study participants recommended that a future study should target the wider population due to the benefits of the intervention. They also suggested that investigators provide healthy food, which can also be through initiating a community farming/gardening project, and provision of blood pressure machines.

### Fidelity

We comprehensively assessed fidelity using four domains of adherence, dosage, quality of delivery, and participant responsiveness – this aligns with contemporary frameworks of fidelity emphasising implementation integrity and participant engagement [60,61]. Overall fidelity was high in all four domains, indicating great confidence that the observed outcomes are attributed to the intervention as designed. Regarding adherence, all three intervention components were delivered as planned. Participant- drop-off and protocol deviations have been reported as common threats to adherence by Slaughter et al [61]; however, our intervention demonstrated strong procedural compliance as noted by full attendance at scheduled educational sessions, weekly calls completion, and receipt of household follow-up. With respect to dosage, the duration of both educational sessions remained in the pre-set acceptable ranges. Our pre-set ranges allowed for adaptive fidelity, thus enabling responsiveness to participant engagement without compromising core intervention components. Literature in implementation science supports flexibility in intervention duration flexibility because it allows for contextual responsive to balance with standardisation [61].

The quality of content delivery was enhanced by the use of standard intervention materials to ensure consistency and the presence of the primary author for direct oversight and real-time observation of delivery integrity. The primary author did not observe any deviations of planned content during delivery. Finally, participant responsiveness wa impressively high as seen by successful weekly calls to all participants, continued engagement in telephonic calls, and no refusal to engage in calls. The exceptionally high responsiveness is suggestive of high acceptability and feasibility, as well as relevant to the participants’ needs. The collective findings of high fidelity in this context provides reassurance that HYPHEN can be rolled out without divergence to original components.

### Strengths and limitations

As strengths, this study is based on an intervention that was informed by underpinning concepts and theoretical approaches such as behaviour change techniques, frameworks, and models, as well as using various sources for co-production (hypertensive study participants, health care workers, community members). To ensure integrity in meaning, this study was conducted in participants’ home language, which is XiTsonga. To further enhance trust, we employed locally trained field workers who come from the local villages and who understand the context. This consideration was essential in gathering rich, quality data from study participants as most adults in the study sample could only speak XiTsonga and they could relate with field workers who came from similar backgrounds. Some limitations were also noted; although we sampled participants from all 32 villages in Agincourt sub-district, from a cohort of adults aged 40 + years with hypertension, we did not employ any stratification during participant sampling which might have led to over- or under-representation from certain villages. We did not collect data on nature of employment as well as clinical history of study participants which could have provided a richer explanation of the outcomes.

### Recommendations for larger trials

Although deemed feasible and acceptable, some amendments are required to the intervention procedures that were carried out before a full trial can be rolled out. Future definite trials should be clear from the onset about the resources that the intervention shall not provide, for example, food parcels, money to buy food, and exercise equipment. We recommend that future trials provide low-sodium salt to participants with a view to longer-term sustainability. A larger clinical trial should record the type of employment (including self-employment) as it appears that many participants may be engaged in self-employment which may affect intervention uptake. Any co-morbodities and clinical history of study participants should be documented and monitored during the clinical trial. Participants who will be included in the clinical trial should have a confirmed diagnosis of hypertension, confirmed by a healthcare worker. Where possible, recruitment should happen at the clinic level.

### Recommendations for policy

We call for stricter regulations of sodium levels in products sold at rural supermarkets. Where possible, community gardening projects should be initiated that promote high intake of fruits, vegetables, and potassium-rich food. Community gardens also promote physical activity. South Africa has approved the National Health Insurance as part of a strategy to strengthen the health system through universal health coverage. Once proven effective, there is an opportunity for HYPHEN to be incorporated in the package of care for hypertension control in similar rural South African communities.

## Conclusion

This was, to the authors’ knowledge, the first study in rural South Africa to determine whether adaptations in physical activity and diet based on existing daily routine would be feasible and acceptable by adults for the control of hypertension. The consequences of uncontrolled hypertension can be devastating and often lead to long-term disability or death. For people living in rural areas of South Africa, such as Agincourt sub-district, it is important to design interventions that will suit their everyday life. This study followed an iterative process of intervention design that was rooted in the contextual reality of adults in a rural setting. Our study was deemed feasible to conduct a larger trial, although certain amendments are noted before the larger trial can be rolled out. Health workers and health promotors in rural South Africa must work together to establish best approaches to implementing physical activity and dietary approaches in their various settings which may be (slightly) different from our study settings.
